# Effectiveness of the adapted bivalent mRNA COVID-19 vaccines against hospitalisation in individuals aged ≥ 60 years during the Omicron XBB lineage-predominant period: VEBIS SARI VE network, Europe, February to August, 2023

**DOI:** 10.2807/1560-7917.ES.2024.29.3.2300708

**Published:** 2024-01-18

**Authors:** Liliana Antunes, Clara Mazagatos, Iván Martínez-Baz, Verónica Gomez, Maria-Louise Borg, Goranka Petrović, Róisín Duffy, François E Dufrasne, Ralf Dürrwald, Mihaela Lazar, Ligita Jancoriene, Beatrix Oroszi, Petr Husa, Jennifer Howard, Aryse Melo, Francisco Pozo, Gloria Pérez-Gimeno, Jesús Castilla, Ausenda Machado, Aušra Džiugytė, Svjetlana Karabuva, Margaret Fitzgerald, Sébastien Fierens, Kristin Tolksdorf, Silvia-Odette Popovici, Auksė Mickienė, Gergő Túri, Lenka Součková, Nathalie Nicolay, Angela MC Rose, on behalf of the European Hospital Vaccine Effectiveness Group

**Affiliations:** 1 Epiconcept, Paris, France; 2 National Centre for Epidemiology, Institute of Health Carlos III, Madrid, Spain; 3 Consortium for Biomedical Research in Epidemiology and Public Health (CIBERESP), Madrid, Spain; 4 Instituto de Salud Pública de Navarra – IdiSNA, Pamplona, Spain; 5 CIBER Epidemiología y Salud Pública, Madrid, Spain; 6 Epidemiology Department, National Health Institute Doutor Ricardo Jorge, Lisbon, Portugal; 7 Infectious Disease Prevention and Control Unit (IDCU), Health Promotion and Disease Prevention, Msida, Malta; 8 Croatian Institute of Public Health, Zagreb, Croatia; 9 Health Service Executive-Health Protection Surveillance Centre (HPSC), Dublin, Ireland; 10 National Influenza Centre Laboratory of Viral Diseases, Sciensano, Brussels, Belgium; 11 National Reference Centre for Influenza, Robert Koch Institute, Berlin, Germany; 12 Cantacuzino National Military-Medical Institute for Research and Development, Bucharest, Romania; 13 Clinic of Infectious Diseases and Dermatovenerology, Institute of Clinical Medicine, Medical Faculty, Vilnius University, Lithuania; 14 National Laboratory for Health Security, Epidemiology and Surveillance Centre, Semmelweis University, Budapest, Hungary; 15 University Hospital Brno, Masaryk University, Brno, Czechia; 16 Infectious Diseases Department, National Health Institute Doutor Ricardo Jorge, Lisbon, Portugal; 17 National Centre for Microbiology, Institute of Health Carlos III, Madrid, Spain; 18 University Hospital Centre Split, Split, Croatia; 19 Service Epidemiology of Infectious Diseases, Sciensano, Brussels, Belgium; 20 Department for Infectious Disease Epidemiology, Robert Koch Institute, Berlin, Germany; 21 National Institute of Public Health, National Centre for Communicable Diseases Surveillance and Control, Bucharest, Romania; 22 Department of Infectious Diseases, Lithuanian University of Health Sciences, Kaunas, Lithuania; 23 European Centre for Disease Prevention and Control (ECDC), Stockholm, Sweden; 24 Members of the European Hospital Vaccine Effectiveness Group are listed under Acknowledgements

**Keywords:** Vaccine effectiveness, COVID-19, SARS-CoV-2, COVID-19 bivalent vaccines, XBB, Hospitalisation, SARI, Europe

## Abstract

We conducted a multicentre hospital-based test-negative case–control study to measure the effectiveness of adapted bivalent COVID-19 mRNA vaccines against PCR-confirmed SARS-CoV-2 infection during the Omicron XBB lineage-predominant period in patients aged ≥ 60 years with severe acute respiratory infection from five countries in Europe. Bivalent vaccines provided short-term additional protection compared with those vaccinated > 6 months before the campaign: from 80% (95% CI: 50 to 94) for 14–89 days post-vaccination, 15% (95% CI: −12 to 35) at 90–179 days, and lower to no effect thereafter.

The European Medicines Agency (EMA) authorised four adapted bivalent mRNA COVID-19 vaccines for use against COVID-19 in September/October 2022: Comirnaty (BNT162b2; Pfizer-BioNTech) and Spikevax (mRNA-1273; Moderna) Original/Omicron BA.1 and Original/Omicron BA.4–5 [1]. During autumn 2022, all European Union/European Economic Area (EU/EEA) countries had vaccination campaigns in place to administer a booster dose, with several countries using the adapted bivalent vaccines [2]. The Omicron-descendent XBB lineage and XBB.1.5 sub-lineage became variants of interest in March 2023 [3]. We estimated the effectiveness of the COVID-19 bivalent vaccines against hospitalisation with PCR-confirmed SARS-CoV-2 infection among patients aged ≥ 60 years with severe acute respiratory infection (SARI) during the XBB lineage-predominant period.

## Vaccine effectiveness study design and patient selection

**Figure 1:**
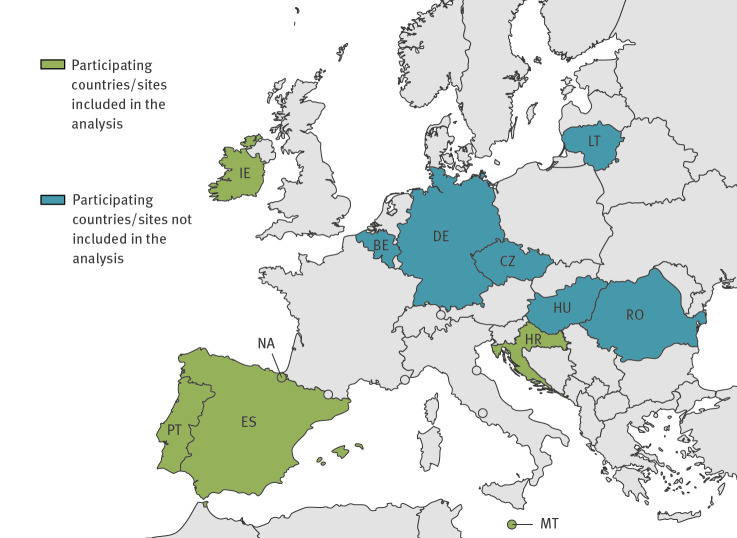
Countries and sites participating in the VEBIS SARI VE network, Europe, 2023

SARI: severe acute respiratory infection; VE: vaccine effectiveness; VEBIS: Vaccine Effectiveness, Burden and Impact Studies.

The methodology of the Vaccine Effectiveness, Burden and Impact Studies (VEBIS) project hospital vaccine effectiveness (VE) study has been described elsewhere [4]. It is a hospital-based, multicentre, case–control study with a test-negative design, including > 50 hospitals at 12 sites in 11 participating European countries (two sites in Spain) (Figure 1) [4]. 

Twelve participating sites: Belgium (BE), Croatia (HR), Czechia (CZ), Germany (DE), Hungary (HU), Ireland (IE), Lithuania (LT), Malta (MT), Portugal (PT), Romania (RO), Spain - 11 regions (ES), Spain - Navarre region (NA). Included in this analysis: ES, HR, IE, MT, NA and PT.

Patients with SARI were individuals hospitalised for ≥ 24 h with at least one of the following symptoms: fever, cough, shortness of breath or sudden onset of anosmia, ageusia or dysgeusia [5]. Cases and controls were SARI patients that tested positive and negative for SARS-CoV-2 by PCR, respectively, within 48 h of admission or in the previous 14 days. 

The XBB lineage-predominant period was defined for each country as the timeframe when the proportion of XBB lineage or XBB.1.5 or XBB.1.5 + F456L sub-lineages among sequenced samples reported to Global Initiative on Sharing All Influenza Data (GISAID) or to The European Surveillance System (TESSy) [6] was above 60%. The final study period comprised records between 15 February and 31 August 2023. Exclusion criteria and the restriction flowchart are available in Supplementary Figure S1.

## SARI patient description

During our study period, we included 743 cases and 3,045 controls aged ≥ 60 years, from 31 European hospitals, in six participating study sites (Figure 1).

Of the total, 70% of cases (n = 518) and 66% of controls (n = 2,012) were vaccinated with a bivalent booster, while 30% (n = 225) of cases and 34% (n = 1,033) controls had not received a bivalent booster but had at least one monovalent vaccine more than 6 months before the start of the bivalent vaccines roll-out (Table 1). Among SARI patients vaccinated with a bivalent vaccine, 90% of cases (n = 466) and 87% of controls (n = 1,746) had received two booster doses. Among SARI patients that did not receive a bivalent vaccine, 82% of cases (n = 184) and 81% of controls (n = 833) had received one booster dose (Table 1). Seventy-three percent of cases (n = 377) and 59% of controls (n = 1,181) with a bivalent booster during the XBB lineage period were vaccinated more than 180 days before symptom onset (Table 1). The median time since vaccination for those vaccinated with a bivalent booster was 215 (IQR: 176–274) days for cases and 193 (IQR: 154–241) days for controls (Table 1).

**Table 1:** SARI patient characteristics by case and control status, VEBIS SARI VE network, Europe, 15 February–31 August 2023 (n = 3,788)

Characteristics	SARS-CoV-2 cases (n = 743)		Test-negative controls (n = 3,045)	
	n	%	n	%
**Age group (years)**				
60–79	280	38	1,536	50
≥ 80	463	62	1,509	50
Median (IQR)	82 (75–88)		79 (72–87)	
**Sex **				
Male	384	52	1,567	51
Female	359	48	1,478	49
**Any chronic condition^a^**				
Yes	596	80	2,438	80
No	147	20	607	20
**Any severe outcome^b^**				
Yes	55	12	198	10
No	388	88	1,699	90
Missing	300	40	1,148	38
**Vaccination status and dose at time of symptom onset**				
**Bivalent booster (received during the bivalent vaccination campaign)**				
Total	518	70	2,012	66
- First booster	18	3	87	4
- Second booster	466	90	1,746	87
- Third/fourth booster	34	7	179	9
**Monovalent vaccine (> 6 months before the start of the bivalent vaccination campaign)**				
Total	225	30	1,033	34
- Full primary course	31	14	167	16
- First booster	184	82	833	81
- Second booster	10	4	33	3
**Days since last bivalent booster dose at time of symptom onset **				
14–89 days	5	1	89	4
90–179 days	136	26	742	37
180–269 days	236	46	892	44
270–359 days	141	27	289	14
Median (IQR)	215 (176–274)		193 (154–241)	

IQR: inter-quartile range; SARI: severe acute respiratory infection; VE: vaccine effectiveness; VEBIS: Vaccine Effectiveness, Burden and Impact Studies.

^a^ At least one of five commonly collected conditions, i.e. diabetes, heart disease, lung disease, asthma and immunodeficiency.

^b^ Admission to an intensive care unit (ICU), use of respiratory support such as mechanical ventilation or extra-corporeal membrane oxygenation (ECMO) or death.

## Effectiveness of bivalent COVID-19 mRNA vaccines

The number of doses and the last vaccination date were used as a proxy to identify the vaccine valency (bi- or monovalent), based on the introduction date of the bivalent vaccines provided by each country (data not shown).

We estimated relative VE (rVE) and incremental VE (iVE), where we applied different vaccination status definitions for the assessment of the vaccine effectiveness (Table 2). We decided not to use never-vaccinated individuals as a reference group, as they have become a smaller group over time, and were not eligible to receive a booster dose during the bivalent vaccine campaign. Patients vaccinated 1–13 days before symptom onset were excluded. Effectiveness was analysed by time since vaccination (TSV) using 60- and 90-day bands.

**Table 2:** Definition of vaccine effectiveness indicators estimated in this study, VEBIS SARI VE network, Europe, 15 February–31 August 2023

Vaccine effectiveness indicator	Vaccinated with bivalent vaccine (‘vaccinated’)^a^	Not vaccinated with bivalent vaccine (‘unvaccinated’)^b^
Relative vaccine effectiveness (rVE)	Vaccinated with any bivalent^a^ booster dose	Vaccinated with at least primary series of vaccination^b^, received > 6 months before the bivalent campaign began
Incremental vaccine effectiveness (iVE)	Vaccinated with primary series vaccination plus two booster doses, with the second booster being a bivalent^a^ vaccine	Vaccinated with primary series of vaccination plus one monovalent booster dose^b^, received > 6 months before the bivalent campaign start

SARI: severe acute respiratory infection; VE: vaccine effectiveness; VEBIS: Vaccine Effectiveness, Burden and Impact Studies.

^a^ Any adapted bivalent mRNA COVID-19 vaccine (Comirnaty (BNT162b2; Pfizer-BioNTech) and Spikevax (mRNA-1273; Moderna) Original/Omicron BA.1 and Comirnaty and Spikevax Original/Omicron BA.4–5). Any booster dose received after the introduction of the bivalent vaccines in each country was considered to be a bivalent COVID-19 vaccine.

^b^ Any monovalent COVID-19 vaccine. Any dose received before the introduction of the adapted bivalent mRNA COVID-19 vaccines in each country were considered to be a monovalent COVID-19 vaccine.

We estimated the odds ratio (OR) of vaccination using a logistic regression model adjusted for date of symptom onset, study site, sex, age and presence of a chronic condition. We carried out a complete case analysis. The VE was calculated as (1−OR) x 100%. Estimates were not shown if there were fewer than 20 vaccinated patients, fewer than five vaccinated/unvaccinated cases or controls, or when the estimate had an absolute difference > 10% from that found from using penalised logistic regression (to assess small sample bias).

**Figure 2:**
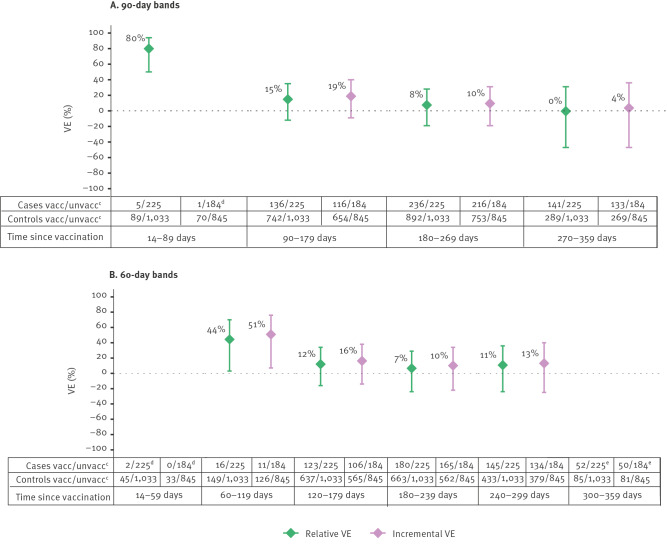
Bivalent COVID-19 relative^a^ and incremental^b^ vaccine effectiveness against hospitalisation among SARI patients aged ≥ 60 years by time since vaccination (A) 90-day bands and (B) 60-day bands, VEBIS SARI VE network, Europe, XBB lineage predominant period, 15 February–31 August 2023

SARI: severe acute respiratory infection; VE: vaccine effectiveness; VEBIS: Vaccine Effectiveness, Burden and Impact Studies.

^a^ Relative VE (rVE) compared those vaccinated with any bivalent booster dose (‘vaccinated’) with those not vaccinated with a bivalent vaccine but vaccinated with at least primary series vaccination, received > 6 months before the bivalent campaign start (‘unvaccinated’). Vaccine valency (bi- or monovalent) was defined based on the date of the introduction of the bivalent vaccines in each country.

^b^ Incremental VE (iVE) compared those vaccinated with primary series vaccination plus two booster doses, with the second booster being a bivalent vaccine (‘vaccinated’), with those vaccinated with primary series vaccination plus one monovalent booster dose, received > 6 months before the bivalent campaign start (‘unvaccinated’). Vaccine valency (bi- or monovalent) was defined based on the date of the introduction of the bivalent vaccines in each country.

^c^ Numbers in the table represent the number of vaccinated/unvaccinated patients for cases and controls for each VE indicator.

^d^ VE estimate not shown:  < 20 vaccinated patients or < 5 vaccinated/unvaccinated cases or controls.

^e^ VE estimate not shown: Indication of small sample bias (estimate had an absolute difference greater than 10% from that found from using penalised logistic regression).

Using 90-day bands, rVE was 80% (95% confidence interval (CI): 50 to 94) 14–89 days post-vaccination with a bivalent vaccine booster dose, 15% (95% CI: −12 to 35) at 90–179 days, 8% (95% CI: −19 to 28) at 180–269 days and 0% (95% CI: −47 to 31) at 270–359 days (Figure 2A). Using 60-day bands, rVE was 44% (95% CI: 3 to 70) 60–119 days post-bivalent dose vaccination, 12% (95% CI: −16 to 34) at 120–179 days, 7% (95% CI: −24 to 29) at 180–239 days and 11% (95% CI: −24 to 36) at 240–299 days (Figure 2B). Small sample size precluded VE estimates for 14–59 and 300–359 days since vaccination. Similar results were found for iVE, for both 60- and 90-day bands of time since vaccination (Figures 2A and 2B).

## Discussion

Our results suggest that the adapted bivalent mRNA COVID-19 vaccines conferred additional protection during the XBB-predominant period compared with those vaccinated with at least primary series vaccination more than 6 months before the bivalent vaccination campaign. We observed a decline in effectiveness, from 80% rVE in the first 89 days to 15% at 90–179 days, and no effect at 270–359 days. Similar results were found for iVE. This is likely due to the overlap of the study populations, as 87% of those who received a bivalent booster had received this as their second booster dose, and 80% of those vaccinated more than 6 months before the start of the campaign had only received a first booster dose of a monovalent COVID-19 vaccine.

The decline of bivalent VE over time against hospitalisation during the XBB period has also been reported by other studies [7-9]. Our VE estimates are consistent with their results, with slightly higher VE point estimates for the more recent vaccinations (up to 119 days). Our study had, however, a smaller sample size for the shorter time since vaccination.

It is challenging to disentangle waning immunity from changes in viral circulation as well as from depletion of susceptible individuals. Although restricting the analysis to the XBB-predominant period, the proportion of XBB-related sub-lineages increased over time, being the lowest at the start, with the underlying XBB sub-lineage also varying over time (XBB, XBB.1.5 and XBB.1.5 + F456L). Five sites sequenced 274 (31%) SARS-CoV-2-positive samples during the analysis period and, of these, 88% were identified as XBB.

Previous results from our VEBIS SARI VE network for a monovalent booster during the Omicron-dominant period showed ≥ 70% VE up until 120 days in those aged ≥ 60 years [10]. Since vaccines were not administered in the same period, it is difficult to make direct comparisons. In addition to different virus circulation, the immunological landscape and exposure risk of the population has also changed over time, with the lifting of non-pharmaceutical measures previously in place and with a high primary series vaccination coverage during our study period [11]. The findings from our analysis should be interpreted in the context of this underlying immunity as the additional protection provided by the bivalent vaccination.

Our study has limitations. Firstly, the autumn 2022 bivalent vaccination campaign took place roughly 6 months before the predominance of XBB in participating countries, reflected in the long median time since vaccination in both cases and controls and in the small sample size for VE estimates for those with more recent vaccinations. Additionally, patient recruitment decreased during the summer, following the decrease of SARI incidence, reflected in the relatively small sample size during the XBB-dominated period. Secondly, we did not adjust for previous SARS-CoV-2 infection, as this is not collected by all sites. This could lead to underestimation of VE, if prior infection is negatively associated with vaccination e.g. if the recently infected are less likely or ineligible to be vaccinated. However, some studies have reported no differences when controlling for previous infection [12]. Thirdly, the analyses were conducted assuming that (i) all booster doses taken after the roll-out of the bivalent vaccines in each country were either bivalent Original/Omicron BA.1 or Original/Omicron BA.4–5; and (ii) the COVID-19 variant causing the infection and subsequent hospitalisation were XBB (XBB, XBB.1.5 or XBB.1.5 + F456L) based on time when these sub-lineages predominated; introducing risk of misclassification of both outcome and exposure of interest.

There are many strengths of our multicentre study. We are able to include data from several countries and sites, which allows us to have a larger sample size and to cover a diverse population across Europe, to have a pooled VE estimate that might be more generalisable. In addition, sites participating in the network follow a generic protocol, which helps to mitigate potential sources of heterogeneity.

## Conclusions

The findings of our study suggest that the bivalent vaccines provided short-term additional protection against hospitalisation among those aged ≥ 60 years during the XBB predominant period.

## Supplementary Data

301000 Supplement

## References

[1] European Medicines Agency (EMA). Authorised COVID-19 vaccines. Amsterdam: EMA. [Accessed: 2 Nov 2023]. Available from: https://www.ema.europa.eu/en/human-regulatory/overview/public-health-threats/coronavirus-disease-covid-19/covid-19-medicines

[2] European Centre for Disease Prevention and Control (ECDC). Overview of the implementation of COVID-19 vaccination strategies and deployment plans in the EU/EEA. Stockholm: ECDC; 2023. Available from: https://www.ecdc.europa.eu/sites/default/files/documents/Overview-vaccination-strategies-COVID-19-8-September-2022.pdf

[3] European Centre for Disease Prevention and Control (ECDC). ECDC de-escalates BA.2, BA.4 and BA.5 from its list of variants of concern. Stockholm: ECDC; 2023. Available from: https://www.ecdc.europa.eu/en/news-events/ecdc-de-escalates-ba2-ba4-and-ba5-its-list-variants-concern

[4] European Centre for Disease Prevention and Control (ECDC). Core protocol for ECDC studies of COVID-19 vaccine effectiveness against hospitalisation with severe acute respiratory infection, laboratory-confirmed with SARS-CoV-2 or with seasonal influenza - Version 2.0. Stockholm: ECDC. 2023. Available from: https://www.ecdc.europa.eu/en/publications-data/core-protocol-ecdc-studies-covid-19-vaccine-effectiveness-against-0

[5] Peralta-Santos A. Assessment of COVID-19 surveillance case definitions and data reporting in the European Union. Briefing requested by the ENVI committee. Brussels: European Parliament; 2020. Available from: http://www.europarl.europa.eu/RegData/etudes/BRIE/2020/652725/IPOL_BRI(2020)652725_EN.pdf

[6] European Centre for Disease Prevention and Control (ECDC). Data on SARS-CoV-2 variants in the EU/EEA. Stockholm: ECDC; 2023. Available from: https://www.ecdc.europa.eu/en/publications-data/data-virus-variants-covid-19-eueea

[7] Fabiani M Mateo-Urdiales A Sacco C Rota MC Fotakis EA Petrone D Relative effectiveness of bivalent Original/Omicron BA.4-5 mRNA vaccine in preventing severe COVID-19 in persons 60 years and above during SARS-CoV-2 Omicron XBB.1.5 and other XBB sublineages circulation, Italy, April to June 2023. Euro Surveill. 2023;28(32):2300397. 10.2807/1560-7917.ES.2023.28.32.2300397 37561053 PMC10416574

[8] Kirsebom FCM Harman K Lunt RJ Andrews N Groves N Abdul Aziz N Vaccine effectiveness against hospitalisation estimated using a test-negative case-control study design, and comparative odds of hospital admission and severe outcomes with COVID-19 sub-lineages BQ.1, CH.1.1. and XBB.1.5 in England. Lancet Reg Health Eur. 2023;35(Oct):100755. 10.1016/j.lanepe.2023.100755 38115965 PMC10730318

[9] Lin DY Xu Y Gu Y Zeng D Sunny SK Moore Z . Durability of bivalent boosters against Omicron subvariants. N Engl J Med. 2023;388(19):1818-20. 10.1056/NEJMc2302462 37043647 PMC10120009

[10] Rose AM Nicolay N Sandonis Martín V Mazagatos C Petrović G Baruch J Vaccine effectiveness against COVID-19 hospitalisation in adults (≥ 20 years) during Omicron-dominant circulation: I-MOVE-COVID-19 and VEBIS SARI VE networks, Europe, 2021 to 2022. Euro Surveill. 2023;28(47):2300187. 10.2807/1560-7917.ES.2023.28.47.2300187 37997665 PMC10668256

[11] European Centre for Disease Prevention and Control (ECDC). Interim public health considerations for COVID-19 vaccination roll-out during 2023. Stockholm: ECDC; 2023. Available from: https://www.ecdc.europa.eu/en/publications-data/interim-public-health-considerations-covid-19-vaccination-roll-out-during-2023

[12] Kirsebom FCM Andrews N Stowe J Ramsay M Lopez Bernal J . Duration of protection of ancestral-strain monovalent vaccines and effectiveness of bivalent BA.1 boosters against COVID-19 hospitalisation in England: a test-negative case-control study. Lancet Infect Dis. 2023;23(11):1235-43. 10.1016/S1473-3099(23)00365-1 37453440

