# Authors’ correction for Euro Surveill. 2024;29(3)

**DOI:** 10.2807/1560-7917.ES.2024.29.8.240222c

**Published:** 2024-02-22

**Authors:** Eurosurveillance editorial team

**Affiliations:** 1 European Centre for Disease Prevention and Control (ECDC), Stockholm, Sweden

In the article entitled ‘Effectiveness of the adapted bivalent mRNA COVID-19 vaccines against hospitalisation in individuals aged ≥  60 years during the Omicron XBB lineage-predominant period: VEBIS SARI VE network, Europe, February to August, 2023’ by Antunes et al., published on 18 January 2024, the number of unvaccinated controls for calculation of incremental vaccine effectiveness (iVE) in Figure 2 was incorrectly reported as 845. The denominator was corrected to 833 and estimates were updated at the request of the authors on 22 February 2024.

