# Supplementary material for: Effectiveness of the adapted bivalent mRNA COVID-19 vaccines against hospitalisation in individuals aged ≥ 60 years during the Omicron XBB lineage-predominant period: VEBIS SARI VE network, Europe, February to August, 2023
Source: Euro Surveill. 2024 Jan 18;29(3):2300708. doi: 10.2807/1560-7917.ES.2024.29.3.2300708 (PMC10797659; doi:10.2807/1560-7917.ES.2024.29.3.2300708)

This supplementary material is hosted by *Eurosurveillance* as supporting information alongside the article "Effectiveness of the adapted bivalent mRNA COVID-19 vaccines against hospitalisation in individuals aged ≥ 60 years during the Omicron XBB lineage-predominant period: VEBIS SARI VE network, Europe, February to August, 2023", on behalf of the authors, who remain responsible for the accuracy and appropriateness of the content. The same standards for ethics, copyright, attributions and permissions as for the article apply. Supplements are not edited by *Eurosurveillance* and the journal is not responsible for the maintenance of any links or email addresses provided therein.

Supplementary Figure S1: Exclusion flowchart

After excluding 11 089 records for patients with missing information to determine inclusion (24 missing consent information and 1523 missing information on SARI case definition), 4967 did not meet the ECDC SARI case definition. Out of these, 31 663 were outside the analysis period. A total of 8 034 records remained.

Further 3 509 were excluded from this analysis: 2 679 in an ineligible population group (2337 age < 60 years, 9 healthcare workers and 333 residents in a long-term care facility), 659 missing key covariates for analysis (14 missing swab date, 21 missing admission date, 70 missing symptom onset date, 135 missing age, 10 missing sex, 63 missing information on common chronic diseases (diabetes, heart disease, lung disease, asthma and immunodeficiency), 124 missing vaccination status and 222 missing date of last received vaccine dose), 168 ineligible due to timing of onset, swab and hospitalisation (165 swabbed > 10 days after symptom and 3 swabbed > 3 days before onset), 3 with ineligible vaccination status (2 with last vaccine dose <14 days before symptom onset and 1 with contra indications for vaccine uptake). A total of 150 records were excluded from one of the countries having data quality issues. A total of 4 375 records remained from 10 sites.

After defining the exposure of interest, 483 records were excluded due to being either never-vaccinated, partially vaccinated, or vaccinated within 6 months prior to start of the bivalent campaign. Three countries were also excluded due to less than 10 cases/controls during the study period (a total of 78 records) and another country was excluded due to having no vaccinated patients (26 records).

A total of 4 246 patients were excluded within the analysis period. The final sample size included 3 788 SARI patients from 6 sites (Spain, Croatia, Ireland, Malta, Navarre, and Portugal).

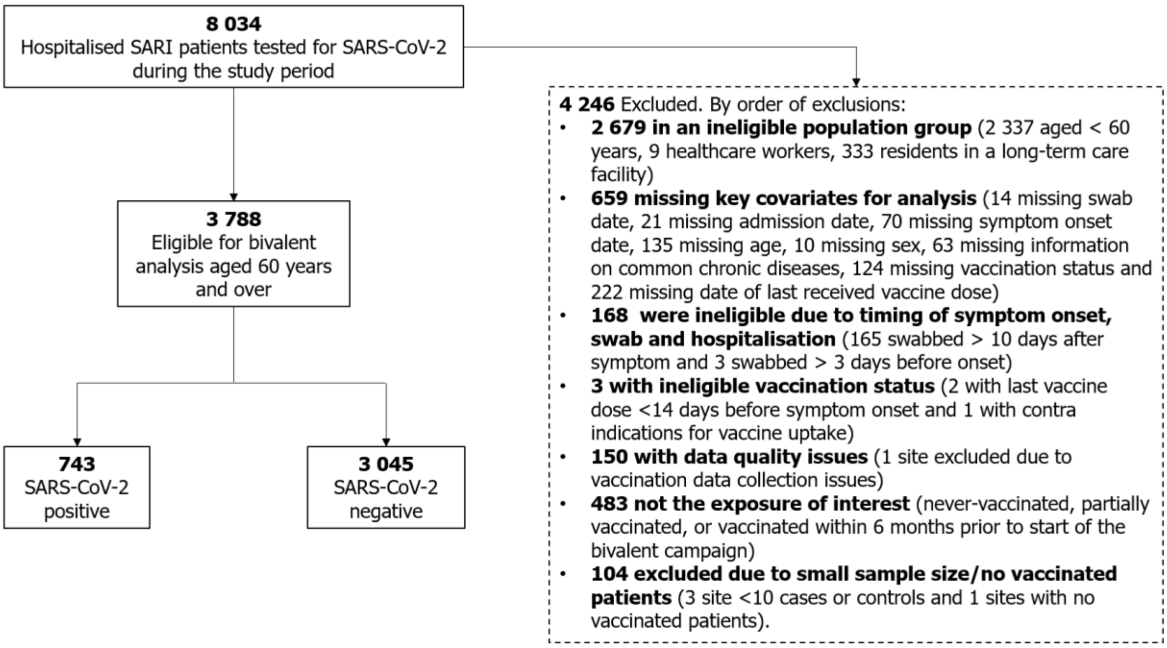

Supplement: Supplement [file 23-00708_ANTUNES_Supplement.pdf]
